# The Medical Association of Georgia

**Published:** 1884-10

**Authors:** Henry Gaither

**Affiliations:** Oxford, Ga.


### THE MEDICAL ASSOCIATION OF GEORGIA.

BY HENRY GAITHER, M. D., OXFORD, GA.

That this organization is beneficial to the medical profession, and consequently to the public, no thoughtful man can doubt. All proper measures adding to its membership extend its benefits to both parties. Now, in brief, what does its history teach? Certainly this. In its early days the door was opened wide, and terms of membership made easy. Consequently its number increased largely from town and country; rich and poor, writers and speakers, listeners and learners. But a change came. Generous men of ample means and paying practice, unacquainted with the war-crippled condition of their country brethren, but with a laudable ambition to appear well before the public in their Transactions, increased the annual dues to $5. Now, if I am correctly informed, this lessened the membership several hundred. To repair this damage the fee was reduced to $1 for all who could not come, and to $2 for such as enjoyed the double benefit of attending the sessions and receiving the Transactions. Under this change prosperity was gradually returning, when, presto! The fee is again increased, and the worthy country doctor, of large practice and small pay, lingers and hesitates between the mortifying alternative of forfeiting membership or applying the fee to the necessities of his family. These vibrations between $1 and $5 do harm. There are other troubles enough. For even skill, eminence, reputation and large practice interpose difficulties. But these can be obviated by the city doctor, who may call in his neighbor, equally eminent and acceptable, to take charge of his patients for a few days. Not so with his country brother, who has no proxy at hand, and would deserve the censure he would be sure to receive if he were to leave without a suitable substitute. Another point. Railroad facilities are not within easy reach of many of our country brethren, and again, before the war negro practice was certain pay. But now few negroes pay anything. And worse still, their former owners, at least many of them, pay little if anything. The subject is fruitful but I forbear, as I only wish to solve the problem, and bring back prosperity by the following means, namely:

1. Restore the $1 and $2 rule lately repealed.

2. Publish the Transactions in cheap, light form, easily distributed through the mails. Not in heavy, showy, costly binding, as if intended for a parlor ornament.

3. Continue the usual annual notices for dues to delinquents, but without the mortifying, humiliating threat of being deposed from membership in default of payment.

4. I will be one of fifty (or any other number the Association may designate) to pay $5 per annum as dues, for life, or as long as needed.

5. When, from death, resignation, or otherwise, vacancies occur, let volunteers be called for at the next annual session, to fill such vacancies.

6. The generous members who, from time to time, have voted to increase the annual dues from all (including the unwilling and unable absentees who could not vote), will, beyond doubt, readily fall into line as aforesaid volunteers.

7. Let the secretary notify the hundreds of "dropped" members, that they can be honorably restored by paying current annual dues only; the Association not exacting annual dues for the past years when they neither enjoyed the benefit of attending the sessions nor received the Transactions.

8. This increase, including new members, will so add to our numbers as to relieve all financial embarrassment, and after a few years, obviate the necessity of further aid by the volunteer corps.

9. Thus more life will be infused and more interest awakened, and short, useful essays and reports of cases worked, from modest but skillful country physicians, who now rarely furnish anything for the Transactions.

10. Being personally on the line between the more and less favored of the profession. I see and hear things that bring me to the conclusion that if the changes I suggest (or something better) are not made, many more names will be "dropped," and consequently, heavier dues required of those that remain. But under the proposed change, if the indigent cannot attend the sessions, they will, at least, receive the Transactions, and be thus fully compensated for the $1 given for it, and I may add the printer too, furnishing a large edition, can do it cheaper and in better style.

